# Alien Hand Syndrome: Neural Correlates of Movements without Conscious Will

**DOI:** 10.1371/journal.pone.0015010

**Published:** 2010-12-13

**Authors:** Michael Schaefer, Hans-Jochen Heinze, Imke Galazky

**Affiliations:** Department of Neurology, Otto-von-Guericke University Magdeburg, Magdeburg, Germany; The University of Melbourne, Australia

## Abstract

**Background:**

The alien hand syndrome is a striking phenomenon characterized by purposeful and autonomous movements that are not voluntarily initiated. This study aimed to examine neural correlates of this rare neurological disorder in a patient with corticobasal degeneration and alien hand syndrome of the left hand.

**Methodology/Principal Findings:**

We employed functional magnetic resonance imaging to investigate brain responses associated with unwanted movements in a case study. Results revealed that alien hand movements involved a network of brain activations including the primary motor cortex, premotor cortex, precuneus, and right inferior frontal gyrus. Conscious and voluntary movements of the alien hand elicited a similar network of brain responses but lacked an activation of the inferior frontal gyrus. The results demonstrate that alien and unwanted movements may engage similar brain networks than voluntary movements, but also imply different functional contributions of prefrontal areas. Since the inferior frontal gyrus was uniquely activated during alien movements, the results provide further support for a specific role of this brain region in inhibitory control over involuntary motor responses.

**Conclusions/Significance:**

We discuss the outcome of this study as providing evidence for a distributed neural network associated with unwanted movements in alien hand syndrome, including brain regions known to be related to movement execution and planning as well as areas that have been linked to inhibition control (inferior frontal gyrus) and experience of agency (precuneus).

## Introduction

The alien hand syndrome (AHS) is a very rare movement disorder. Patients with AHS experience one of their limbs as alien, which acts autonomously and performs meaningful movements without being guided by the intention of the patient [1], [2], [3]. The patients find themselves unable to stop the alien hand from reaching and grabbing objects without using their other hand. Patients are aware that the limb is still part of their body, but they report the feeling as if an external agent is controlling the limb. Consequently, they often describe it in the third person.

The phenomenon of AHS is complex and has various clinical manifestations, possibly related to different lesion sites. AHS has been reported subsequent to lesions of the supplementary motor area (SMA), anterior cingulate, corpus callosum, anterior prefrontal cortex, posterior parietal cortex, and thalamus [3], [4], [5], [6], [7]. The neural mechanisms of this movement disorder still remain unclear. It has been proposed that unwanted movements may arise because of a release of the primary motor cortex (M1) from conscious control by intentional planning systems [8].

Here we report data of a patient diagnosed with corticobasal degeneration and left hand AHS. His left hand showed relatively preserved volitional motor functions. Although there were spontaneous movements of the alien hand, we also had the possibility to elicit alien movements of the hand in a controlled way. We were able to evoke movements of the hand by slightly pushing the hand away from the patient's body, which then resulted in a small movement into the opposite direction. This behavior is also known as “Gegenarbeiten”, meaning counteracting or working against [5]. Using this reliable behavioural effect we conducted a functional magnetic resonance imaging (fMRI) study to further examine the neural correlates of unconscious or alien movements.

The study consisted out of two fMRI experiments. We first examined unwanted movements the way described above. The second experiment was a motor localizer scan to assess brain areas associated with conscious movements (similar to [8]).

## Methods

### Case report

The study adhered to the Declaration of Helsinki and was approved by the human subjects committee of the Otto-von-Guericke University Magdeburg. The participant gave written, informed consent to participate in the study. The patient has given written consent to the publication of this case report.

The 75-year-old right-handed gentleman (WH) was diagnosed with Parkinson's syndrome five years ago. Within the last six months he reported a rapid loss of control of his left hand. It became much more stiffed and lost fine motor skills. When he walked down a stair he was not able to release the railway voluntary. Playing table tennis became awful. He was not able to serve because the left hand did not loose the grip of the ball. Dopaminergic medication was not as efficient as it used to be at the beginning of disease.

**Figure 1:**
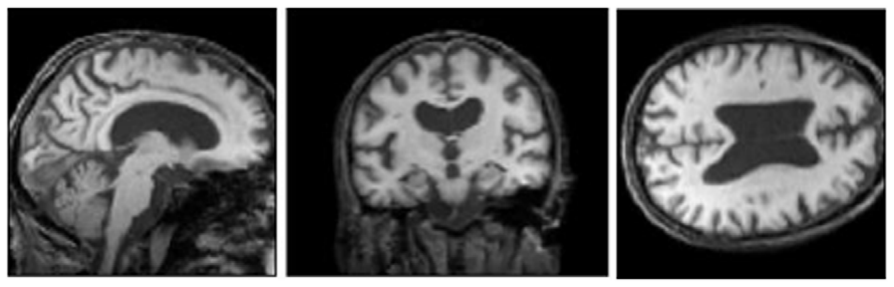
T1-weighted structural MRI of patient WH. MRI shows altered and increased ventricles on both sides. Informed consent was obtained for publication of this figure.

Clinically we saw an uplifted arm and reduced arm swing on the left side, strongly left sided rigidity and intermitted irregular myoclonus of the left arm. There were no signs of sensory deficit; reflexes were obtained symmetrical. Tracer studies (DAT Scan and IBZM Spect) revealed loss of presynaptic dopamine as well as a reduction of the post-synaptic dopaminergic receptor state. Structural MRI showed increased and asymmetrical ventricles (see Fig. 1). Based on the clinic and imaging we diagnosed an atypical Parkinsonian syndrome by possible corticobasal degeneration.

After increasing of dopaminergic medication rigidity improved but by now WH reported attacks of his left hand toward his body: the hand grabbed into his face and he could not loose the grip voluntary. When he used his right hand to release the left hand from his face the grip of the left hand became even stronger and he got scratched. He then controlled his hand during night covering up the left arm and keeping the bedside lamp turned on. Neuropsychological testing revealed intermanual conflict (the left hand did not let go objects), transitive dyspraxia using an object (i.e. hole-puncher), only slightly reduced tactile sensory, and tonic grasping. No mirror movements or synkinesis was observed.

### Functional imaging procedure

The first fMRI experiment aimed to induce alien hand movements. If the alien hand was slightly stimulated, the hand immediately made a small “alien” movement in the opposite direction (“Gegenarbeiten”). If the healthy hand was touched in that way, no reaction could be observed. We made use of this effect in order to establish an fMRI paradigm with highly replicable events. The first experiment consisted of 8 blocks of left hand stimulation and 8 blocks of right hand stimulation (pseudorandomized order). Each block lasted for 16 seconds, followed by breaks of 12 s during which a fixation asterisk was shown. During the presentation of the fixation asterisk the patient was told to relax. In each block an experimenter who attended WH in the scanner room slightly stimulated WH's hand in the way described above. The stimulation was done in a repetitive way (every 2 s).

The second fMRI experiment aimed to examine the cortical representations of willed movements of the alien as well as the healthy hand. To this end we instructed WH to perform voluntary flexion-extension movements of his left or right hand (with a frequency of 2 seconds), alternating with rest periods. The blocks were signaled by visual cues (“Move left hand”, “Move right hand”, or “Rest”). The experiment consisted of 8 blocks of left hand moving and 8 blocks of right hand moving (pseudorandomized order). Each block lasted for 16 seconds, followed by breaks of 12 s during which a fixation asterisk was shown. For both experiments the patients performance was online monitored.

FMRI data were acquired with a 3 T Magnetom Trio Siemens scanner for T2-weighted functional MR images using axially oriented echo-planar imaging (TR = 2 s, TE = 28 ms, flip angle  = 90°, 32 slices, 5 mm thickness). Data were acquired in two scanning sessions. Due to T1 equilibration effects the first four volumes of each session were discarded. For anatomical reference, a T1-weighted anatomical image was obtained (3D-SPGR, TR = 24 ms, TE = 8 ms). Visual instructions (experiment 2) were back-projected onto a screen at the end of the scanner bed close to the subject's feet. The patient viewed the images through a mirror mounted on the birdcage of the receiving coil. In addition to a head strap, foam cushions were placed tightly around the side of the subject's head to minimize head motion. Data preprocessing and statistical analyses were carried out using SPM5 (Statistical Parametric Mapping, Wellcome Department of Imaging Neuroscience, University College London, London, UK). Functional images were realigned to correct for inter-scan movement using sinc interpolation and subsequently were normalized into a standard anatomical space (MNI, Montreal Neurological Institute template) resulting in isotropic 3 mm voxels. Data were then smoothed with a Gaussian kernel of 6 mm full-width half maximum.

Statistical parametric maps were calculated using multiple regression with the hemodynamic response function modeled in SPM5. For data analyses of experiment 1 we used a block-design model with a boxcar regressor convolved with the hemodynamic response function to compare brain responses elicited by stimulation of the alien hand compared with stimulation of the healthy hand. For data analyses of experiment 2 we used an analogue procedure (regressors left hand movements, right hand movements, rest). We report results corrected for multiple comparisons (at p<0.05).

## Results

### Behavioral data

In the first experiment WH showed unwanted movements of the alien hand whenever being stimulated by the experimenter in the scanner room. Stimulation of the healthy hand failed to evoke any movements. No other unwanted movements of the alien hand could be observed by the experimenter inside or outside the scanner room.

In the second experiment (motor localizer) WH performed the task for both hands, but reported afterwards that moving his alien hand required high concentration and effort.

### Imaging data

**Figure 2:**
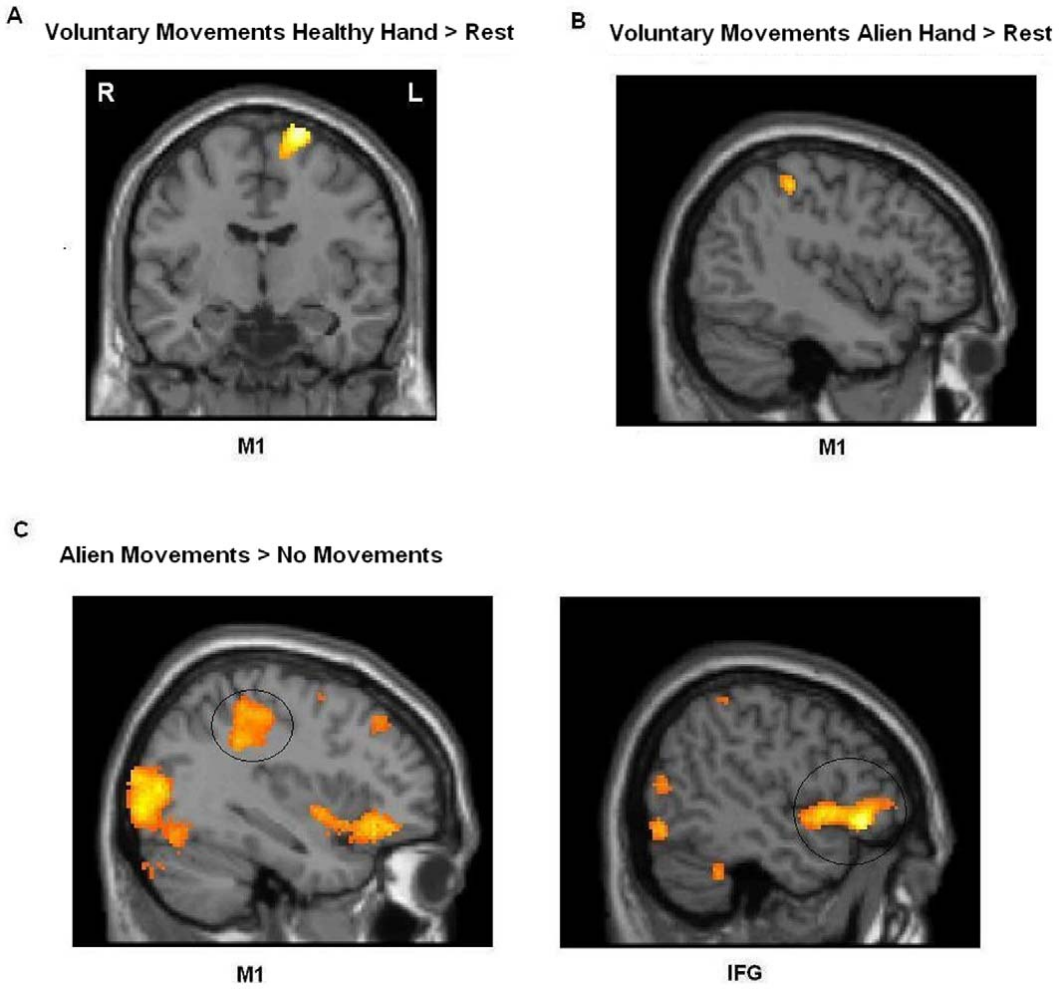
Image of statistic parametric mapping for the contrast (A) voluntary movements of the healthy hand relative to rest. Brain responses showed activation of M1, SMA, and premotor areas when moving the healthy hand. (B) When voluntary moving the alien hand, M1 and other areas showed significant activations (see table 1 for details). The figure shows activation of the contralateral M1. (C) Alien movements could be elicited by experimenter's stimulation (see text for further explanation). The figure demonstrates activation of M1 and IFG when alien movements were evoked (see circles) (relative to stimulation of the healthy hand). Stimulation of the healthy hand revealed no significant brain areas (relative to the stimulation of the alien hand). Areas of significant fMRI signal change are shown as color overlays on the T1-MNI reference brain.

FMRI data of the first experiment revealed that alien movements in patient WH were associated with a network of different brain areas. Stimulation of the left alien hand relative to stimulation of right healthy hand resulted in activation of M1 (36, -38, 50; z = 4.07; p<0.05, family-wise-error (FEW) corrected), left premotor cortex (-56, 4, 42; z = 3.99); precuneus (BA7, 0, -58, 72; z = 6.56), right inferior frontal gyrus (IFG) (50, 30, -6; z = 6.26), and cerebellum (-20, -70, -46; z = 5.88) (see Table 1 and Fig. 2). The contrast right (healthy) hand stimulation relative to left (alien) hand stimulation failed to show any significant activations (p<0.05, corrected) (see Table 1). This was expected because the stimulation of the healthy hand did not yield in any involuntary movements.

**Table 1:** Results of random effects analysis (p<0.05, corrected, L = left hemisphere, R =  right hemisphere).

	contrast	brain region	MNI location (x, y, z)	peak t-value	peakz-value
hand stimulation experiment	left hand > right hand	R M1L premotor cortexR IFGaaaaR superior frontal gyrusprecuneusR parieto-occipital cortexL parieto-occipital cortexventral striatum (putamen)R parietal operculumcerebellumR occiptal cortex	36–38 50–56 4 4250 30–628 20–1414 24 580–58 724–90 46–26–86 42–16 20–8–46–32 20–20–70–4628–90 2	4.164.086.594.695.266.947.205.824.944.716.166.58	4.073.996.264.565.086.566.775.584.744.585.886.25
	right hand > left hand	-	-	-	-
motor localizer experiment	left hand > rest	R M1L M1SMAVMPFCL precuneusR precuneusL parieto-occipital cortexR parieto-occipital cortexcerebellumR occipital cortexL occipital cortex	42–36 52–26–32 540–4 76–2 34 -16–12–52 6816–50 74–12–88 368–86 46–2–46–2216–104–6–14–58 12	3.444.243.455.864.364.254.014.124.794.004.44	3.424.213.435.784.334.223.994.094.743.984.40
	right hand > rest	L M1SMAL premotor cortexcerebellum	–20–14 78–18 2 74–14 0 644–34–42	7.947.536.744.04	7.727.356.614.01

The second experiment aimed to identify brain areas activated by voluntary movements of the hands. Voluntary moving the alien hand in this motor paradigm resulted in significant activation of M1, SMA, ventromedial prefrontal cortex (VMPFC), precuneus, and cerebellum (relative to rest, see Table 1 and Fig. 2). The active brain regions of M1 and precuneus were identical to the brain areas associated with alien movements in the first experiment. For the healthy hand the results revealed significant activations in M1, SMA, premotor cortex and cerebellum (relative to rest). The M1 activation was located relatively superior, probably due to plasticity processes.

## Discussion

This study reports neural correlates of movements without conscious will in a case of AHS. Results of functional imaging revealed a network of different brain areas engaged during unwanted movements, including M1, premotor cortex, parietal cortex (precuneus), right IFG, and cerebellum. M1, premotor cortex, and cerebellum are also known to be involved when performing voluntary movements [9]. In contrast, activation of the precuneus has not been related to motor behavior, but to first-person perspective taking, experience of agency and self-processing operations [10]. Since this brain structure was activated both when alien movements took place as well as during voluntary movements of the alien hand, we suggest that this brain area may reflect a conflict of agency of the alien hand. Although the patient was able to perform the motor task in the localizer experiment, he mentioned that he needed to spend much effort and concentration to execute the conscious movements of the alien hand. Thus, we think that both in the motor localizer task as well as when unwanted movements were elicited by the experimenter, conflicts of agency may have occurred, associated with activation of the precuneus. This interpretation is supported by the lack of precuneus activation for movements of the healthy hand. Furthermore, the study by Assal et al. [8] reported similar activation of parietal areas during unwanted movements as well as voluntary movements of the alien hand.

Similar to the precuneus, the IFG is not typically activated in motor paradigms. This brain area has also been reported to be uniquely activated during alien movements in the fMRI study by Assal et al. [8]. Recent lesion studies discuss a role for the right IFG in inhibitory control over motor responses [11]. Since the IFG was active only in the condition when we elicited involuntary movements, involvement of this brain region may reflect attemptions to control and inhibit movements of the alien hand. Thus, we argue that both precuneus as well as the IFG may reflect conflicts of agency in unconscious and unwanted movements.

However, other explanations may also account for the activation of the right IFG. Since prefrontal areas are also known to be associated with resting state activity [12], brain responses in IFG may also correspond to the fact that in the first experiment the patient did not have to perform a task. Nevertheless, since the results for the healthy hand failed to reveal any significant activation in IFG or other frontal areas, we think it is unlikely that activation in IFG may reflect resting state activity.

The results of the motor localizer experiment revealed different brain activations for the hemispheres (see, for example, the activation of M1 during voluntary movements). These differences seem to reflect reorganization processes associated with the corticobasal degeneration. Those processes may also explain the bilateral activation of M1 when the patient performed voluntary movements with the left hand. Furthermore, the activation in VMPFC and striatum may be linked to an altered striato-frontal connectivity due to Parkinson's disease.

The experimental design of this study made use of a behavioral effect we observed in our patient (“Gegenarbeiten”). Although this enabled us to elicit alien movements in a controlled way, the design may also bear some disadvantages. The slight stimulation of the hand might also have elicited sensorimotor brain regions. Nevertheless, we spent any effort not to passively move the hands and to stimulate both hands in the same way. Since the stimulation procedure for the healthy hand did not reveal any significant brain activations, we think that it is unlikely that the stimulation procedure may have caused the brain activations we report.

Further, we have to consider that the alien hand movements we elicited in the experimental paradigm (“Gegenarbeiten”) are different from the more complex motor behavior we report when introducing the patient (see above). However, since these less complex movements are also not guided by conscious will, we think that they represent “alien” movements similar to the complex ones described above.

Taken together, the results of the current study extend the previous fMRI results of Assal et al. [8] by showing that at least in some cases AHS is associated with a broader network of active brain regions known to be related to movement execution and planning as well as with areas that have been linked to inhibition control (IFG) and experience of agency (precuneus).
